# The association of patient trust and self-care among patients with diabetes mellitus

**DOI:** 10.1186/1471-2296-5-26

**Published:** 2004-11-16

**Authors:** Denise E Bonds, Fabian Camacho, Ronny A Bell, Vanessa T Duren-Winfield, Roger T Anderson, David C Goff

**Affiliations:** 1 Department of Public Health Sciences, Wake Forest University School of Medicine, Winston-Salem, NC, USA; 2 Department of Internal Medicine, Wake Forest University School of Medicine, Winston-Salem, NC, USA; 3 Maya Angelou Center for Minority Health, Wake Forest University School of Medicine, Winston-Salem, NC, USA

## Abstract

**Background:**

Diabetes requires significant alterations to lifestyle and completion of self management tasks to obtain good control of disease. The objective of this study was to determine if patient trust is associated with reduced difficulty and hassles in altering lifestyle and completing self care tasks.

**Methods:**

A cross-sectional telephone survey and medical record review was performed to measure patient trust and difficulty in completing diabetes tasks among 320 medically underserved patients attending diabetes programs in rural North Carolina, USA. Diabetes tasks were measured three ways: perceived hassles of diabetic care activities, difficulty in completing diabetes-related care activities, and a global assessment of overall ability to complete diabetes care activities. The association of patient trust with self-management was examined after controlling for patient demographics, physical functioning, mental health and co-morbidities.

**Results:**

Level of patient trust was high (median 22, possible max 25). Higher trust levels were associated with lower levels of hassles (p = 0.006) and lower difficulty in completing care activities (p = 0.001). Patients with higher trust had better global assessments of overall ability to complete diabetes care activities (p < 0.0001).

**Conclusion:**

Higher patient trust in physicians is associated with reduced difficulty in completing disease specific tasks by patients. Further studies are needed to determine the causal relationship of this association, the effect of trust on other outcomes, and the potential modifiability of trust

## Background

Diabetes mellitus is a disease associated with significant morbidity and mortality [1,2]. Patients with diabetes have higher rates of coronary artery disease, retinopathy, neuropathy and nephropathy [1]. Many of these complications can be prevented with appropriate medical care[3,4] This care, however, often requires significant alterations in lifestyle and strict adherence to self-care tasks, such as checking blood sugars, and taking medications by the patient [5]. Previous research has shown that patients with diabetes and other diseases often have difficulty in adopting lifestyle changes and completing self-care tasks[6]. The cause of this poor adherence is multi-factorial and includes patient characteristics such as educational level, [7-9] treatment regimen characteristics, [10] and characteristics of the clinical setting [11,12]. Patient's perception on the intrusiveness of treatment regimens and their perceived self-efficacy in completing the task have also been demonstrated to affect adherence to diabetes treatment [13,14].

Aspects of the patient-physician relationship such as communication and empathy have been shown to be important to patient's adherence [15-18] and ability to complete self-care tasks [7,19,20]. Patient trust is another component essential to the doctor-patient relationship [21]. Defined variously as a set of beliefs or expectations that the doctor will act in the patient's best interest, or as a reassuring feeling of confidence in the doctor, [22,23] trust provides the foundation for many of the other aspects of the relationship such as communication and empathy [23]. Despite the significance of trust, there has been relatively little research on this aspect of the doctor-patient relationship. Most studies examining trust have focused on characteristics that predict trust levels [24-28] or the influence insurance characteristics have on trust [29,30]. Limited studies have been done examining the relationship between patient trust and medical outcomes. Safran et al found that adherence to physician recommended lifestyle changes was significantly associated with patient trust levels [31]. Higher levels of patient trust have also been associated with lower hemoglobin A1c levels in patients with type 2 diabetes [32]. None have measured trust and its effect on patient's ability in completing self-care tasks.

Given the underlying importance of trust in the doctor-patient relationship and the effect of the doctor-patient relationship on adherence and self-care, we hypothesized that higher levels of patient trust in physicians may be associated with reduced difficulty of self-care tasks. To explore this hypothesis, we conducted a survey of patients with diabetes to determine if level of trust is associated with the outcomes of difficulty and hassles in completing self-care tasks.

## Methods

### Data Source

Project IDEAL (Improving Diabetes Education, Access to Care and Living) was an initiative to increase the quality of care and quality of life for underserved patients with diabetes in North Carolina. Full details of the study have been published elsewhere [33]. Briefly, fourteen clinical sites across North Carolina were funded by the Kate B. Reynolds Charitable Trust to establish programs to improve the quality of care provided to underserved patients with diabetes. Each developed its own unique diabetes intervention. Evaluation of the initiative was conducted by the Wake Forest University School of Medicine Department of Public Health Sciences using both quality of care and quality of life indicators. To assess the effectiveness of the initiative, a random sample of patients from each program was selected for inclusion at each point in time (1998 pre-initiative and 2001 post-initiative). Quality of care was assessed through a chart review of randomly selected patient. Diabetes Quality of Care (DQIP) [34] and Health Plan Employer Data and Information Set (HEDIS) [35] measures were used as standards for quality of care. Quality of life was assessed through a telephone survey of the same patients, allowing both quality of care and quality of life indicators to be linked. The telephone survey was composed of portions of the Medical Outcomes Study Short Form 36 (SF-36) [36], the DQIP Patient Measures [34] and Diabetes-39 Health Related Quality of Life instruments [37]. To determine the relationship between self-management and level of patient trust, an established trust instrument was added to the post intervention survey.

### Trust Instrument

To measure patient trust, we used the Wake Forest University Trust Scale. This scale has been tested in multiple populations and found to be reliable with good construct validity [38,39]. We used the 5 question institutional trust scale as many of the patients were cared for by multiple physicians. This trust measure is a summation of five items that are adapted from a longer trust instrument to ask the patient's view of doctors in general [39]. The items are: "The doctors at 'this clinic' will do whatever it takes to get patients all the care they need;" "The doctors at 'this clinic' are extremely thorough and careful;" "The medical skills of the doctors here are not as good as they should be;" "You have no worries about putting your life in the hands of the doctors at 'this clinic;" "All in all, you trust the doctors at 'this clinic' completely." A five point scale was used with answers ranging from strongly disagree to strongly agree (1–5), with a higher score indicating a higher level of trust.

### Self-Management Measures

Patient self-care measures were taken from the telephone survey. The methods for this survey have been described elsewhere [40]. We examined 3 measures: patients' perceived hassle associated with self-care tasks (hassles), patients' difficulty in completing recommended care activities (difficulty), and a global assessment of their ability to care for their disease (care). Briefly, hassle was measured with a 7-item scale that asked patients to rate how much of a problem or hassle it has been to complete diabetes-related tasks over the past 4 weeks. Items in the scale included remembering to take medication and test blood sugar, making meal plans, avoiding particular foods, having to keep a care schedule in mind, organizing the daily routine around a medical care activity, and total time spent in managing their disease. This scale is part of the DQIP instrument [34]. All questions were measured using a 5-point scale from "no problem" (1) to "a major hassle" (5) with a higher value equalling more hassle. Respondents also had the choice of indicating the question "does not apply". This resulted in a variable number of questions being answered for each patient. To account for this variation, we calculated an average response for each patient by summing the responses and dividing by the number of questions answered by that patient.

Our second outcome, difficulty in completing care activities, was measured in a 5-item scale also taken from the DQIP measures [34]. This scale asked patients about the difficulty they had in completing 5 specific care activities exactly as their doctor suggested. Activities included 1) taking their medication as prescribed, 2) exercising regularly, 3) following their diet, 4) checking their blood sugar, and 5) checking their feet for wounds or sores. Answers were scored on a 5-point scale from "not at all difficult" (1) to "extremely difficult" (5) with an option for "does not apply". Again, to account for variation in the number of questions answered, we calculated an average score, excluding measures that did not apply.

The final adherence measure we examined was a global measure of care. This single item asked patients to rate how they "...are at taking care of their diabetes". Possible responses on a 5-point scale ranged from "I stay right on top of it at all times" (1) to "I let it slip way too much" (5).

### Independent Variables

We considered several modifying factors that might influence the relationship between trust and self-care measures. These factors were selected either because they have been shown to influence patient self-care or because they have been used in previous studies on patient trust and outcomes. Modifying factors were obtained from either the survey or through the chart review. Patient's age, gender, race (white, non-white) and insurance (any, none) were obtained from chart review. A co-morbidity score (none, 1–2, greater than 2) was generated based on the number of additional diabetes-related diseases (hypertension, coronary artery disease, non-traumatic amputation, nephropathy, neuropathy, peripheral vascular disease and smoking) identified during chart review. The Medical Outcomes Study Short Form 36 (MOS SF-36) [36] subscales for physical and mental health were included as part of the patient interview and were used to assess health-related quality of life. The length of the relationship with the health care professional and the number of visits to a provider for diabetes care were obtained from the telephone survey. The use of insulin by the patient was collected from the chart review and was included in the analyses. Imputed values were created for missing measures and analyses were run with and without imputed variables to detect the effect of these variables.

### Statistical Analysis

To determine the association of patient trust and our outcomes of patient hassles, difficulty in completing care activities and global assessment, we used Generalized Estimating Equation[41] methods to account for clustering of patients within IDEAL program sites. A regression analysis using the xtgee command in STATA/SE 7.0 (STATA Corporation, College Station, TX) was performed for each outcome of interest. Models adjusted for age, gender, race, insurance status, number of co-morbidities, insulin use, number of visits for diabetes in the last year, length of relationship with the provider, physical functioning, and mental health.

## Results

Three hundred and twenty-six individuals with diabetes had information from both the telephone survey and chart review portions of the study. The response rate for the telephone survey was 67%.

The mean age of the respondents was 60, over two-thirds were female and forty percent were non-white (see table 1). Most respondents (75%) had some sort of insurance, primarily Medicare or a Medicare HMO. Most (79%) had least one co-morbid condition and the average number of co-morbid diseases was one. Average score on the MOS-SF 36 physical functioning and mental health scale was 57.0 (sd 29.8) and 73.5 (sd 21.2), respectively. Diabetes control was generally good with the average hemoglobin A1c 7.3%, although the range was wide (4.4%–14.6%). Twenty four percent of the sample had insulin listed as a treatment in their medical records. When asked about the length of time they had been seeing a provider or clinic, 15% responded less than 1 year, 21% 1–2 years, and 65% more than 2 years. The average number of visits to a provider for diabetes care in the last 12 months was 3. The level of patient trust in their physician was high, with an average and median level of trust of 22 (sd 3, range 11–25).

**Table 1:** Description of the Sample (n = 326)

Age, mean	60 (sd 12.0, range 19–89)
Gender, female	67% (n = 217)
Race, non-white	40% (n = 120)
Insurance, none	25% (n = 82)
Co-morbidities, any (%)	79% (n = 228)
Co-morbidities, mean (%)	1.1 (sd 1.0, range 0–4)
Hemoglobin A1c, mean	7.3% (sd 1.7, range 4.4–14.6)
Physical Health (max 100)	57.0 (sd 29.8)
Mental Health (max 100)	73.5 (sd 21.0)
Number of years with provider/clinic*	
Less than 1 year	13% (n = 44)
1 to 2 years	19% (n = 62)
More than 2 years	58% (n = 190)
Number of visit in last 12 months	3 (sd 1.0)
Patient Trust (max 25)	22 (sd 3, range 11–25)

* Number does not equal 100% due to participant who responded that they did not have a regular source of diabetes care

When asked about hassles associated with their diabetes, respondents reported the highest degree of hassle with avoiding foods they enjoyed (higher score equals more hassle: mean 2.4, SD 1.4, range 1–5) (table 2). Taking medication (mean 1.2, SD 0.7, range 1–5) and testing blood sugar (mean 1.4, SD 0.9, range 1–5) were the care activities associated with the lowest level of hassle. Similarly, patients reported low levels of difficulty when asked about taking their medication for their diabetes (higher score equals more difficulty: mean 1.2, SD 0.5, range 1–4), while they reported higher levels of difficulty in following a diet for their diabetes (mean 2.1, SD 1.0, range 1–5) and exercising regularly (mean 2.5, SD1.3, range 1–5). Overall, the patients surveyed reported high global assessments of their self care for their diabetes (higher score equals worse self care: mean 2.0, SD 0.9, range 1–5).

**Table 2:** Average score for global outcome measures and individual components of each measure

Outcome Measure	Mean (SD, Range)
Hassles associated with diabetes care*	
Average for entire scale	1.6 (0.7, 1–4.5)
Remembering to take diabetes medicine	1.2 (0.7, 1–5)
Remembering to test blood for sugar	1.4 (0.9, 1–5)
Making meal plans	1.6 (1.2, 1–5)
Avoiding foods you enjoy	2.4 (1.4, 1–5)
Keeping schedule in mind at times	1.7 (1.2, 1–5)
Organizing daily routine around diabetes care	1.4 (0.9, 1–5)
Total time spent managing diabetes	1.5 (1.0, 1–5)
Difficulty in performing self care activities**	
Average of entire scale	1.7 (0.5, 1–3.8)
Taking medications as prescribed	1.2 (0.5, 1–4)
Exercising regularly	2.5 (1.3, 1–5)
Following diet	2.1 (1.0, 1–5)
Checking blood for sugar	1.5 (0.9, 1–5)
Checking feet for wounds or sores	1.2 (0.5, 1–5)
Global assessment of ability to care for diabetes***	2.0 (0.9, 1–5)

* Higher equals more hassles associated with care activity

** Higher equals more difficulty in completing care activities

*** Higher equals lower assessment of ability to care

In bivariate analyses, a higher levels of hassles was associated with younger age (p < 0.001), non-white race (p = 0.03), and lower score for mental health. There was no association with gender, insurance status, co-morbidities, use of insulin, physical health, length of the relationship with the provider, or the number of visits for diabetes in the past year. A higher difficulty in completing care activities was associated with younger age (p = 0.002), female gender (0.003), non-white race (p = 0.03), worse physical health (p < 0.001) and worse mental health (p = 0.001). There was no association with insulin use, co-morbidities, length of relationship, or number of visits in the past year. Better self rated ability to care for diabetes was also associated with older age (p = 0.004), better physical health (p = 0.02), and better mental health (p < 0.0001). There was no association with gender, race, co-morbidities, use of insulin, length of relationship, or number of visits in the past year.

In multivariate regression analysis, level of patient trust was significantly associated with each of the outcomes we examined. Patients with higher levels of trust in their physician reported lower levels of hassles with disease (GEE parameter -0.03, 95% CI -0.05 to -0.008, p = 0.006). Similar finding were seen when self-reported difficulty was examined. Higher level of patient trust was again associated with less reported difficulty in caring for diabetes (GEE parameter -0.02, 95% CI -0.03 to -0.01, p = 0.001). Our final patient outcome, global assessment of ability to care for diabetes, also showed a significant association with higher levels of patient trust associated with higher self-reported ability to care for diabetes (GEE parameter -0.05, 95% CI -0.08 to -0.02, p < 0.0001). Thus, if a patient responded "strongly agree" to all the trust questions rather than "agree" (representing a 5-point increase in level of trust), self-reported hassles would decrease by approximately 0.2 points (about 30% of a standard deviation in hassles). Similarly, for a 5-point increase in trust, self-reported difficulty would decrease by 0.1 points (about 20% of a standard deviation in difficulty) and global ability to care for diabetes would decrease by 0.3 (about 33% of a standard deviation in global ability to care for diabetes). (table 3)

**Table 3:** General estimating equations regression parameters for difference in outcomes associated with a 5-point difference in trust

Outcome	GEE parameter estimates (95% CI)
Hassles associated with diabetes care	-0.2 (-0.3 to -0.05)
Difficulty in performing self care activities	-0.1 (-0.20 to -0.05)
Global assessment of ability to care for diabetes	-0.3 (-0.40 to -0.10)

## Discussion

Diabetes is a disease requiring many types of interventions to prevent the associated morbidity and mortality. These interventions include medications to control elevated glucose levels and finger sticks to check blood sugars. Additionally, significant alterations in lifestyle such as increasing exercise and changing the type of food one eats are an essential portion of the treatment regimen. In our sample of low-income patients with diabetes, we found low reported hassle of completion of diabetes tasks, low reported difficulty in completing diabetes related tasks and good self reported ability to stay on top of their disease. The patients in this study did report more difficulty in making lifestyle changes such as exercising regularly and following recommended diets than taking medications. Other studies examining adherence in diabetic patients have found similar findings. Ruggiero et al, in a nationwide survey of individuals with diabetes, found that over 90% reported always or usually taking their medication but only 64% always or usually followed dietary recommendations and less than half always or usually exercised [6].

Higher levels of trust were associated with lower reported levels of hassles, lower self-reported difficulty in completing care activities, and improved self-reported global ability to take care of diabetes. These results support our hypothesis of increased completion of self-management tasks with higher levels of patient trust in physicians. There are several possible causes for this association. Patients who are actively engaged in their medical care and jointly make health care decisions with their provider may have less difficulty and hassles in performing self-care activities that they had input on. These patients may also have higher trust levels because they have been engaged as an active participant in the health care decision by their provider. Additionally, medical regimens used to treat chronic disease are complicated. Patients may not fully understand the medical rationale behind particular recommendations such as exercise and diet. Additionally, exercise and diet may not result in immediate improvement in symptoms and often cause initial discomfort or feelings of deprivation, thereby providing little positive feedback and reinforcement. Patients who have high levels of trust may be willing to overcome the initial discomfort and maintain faith that these difficult changes will ultimately be beneficial to them. Our results indicate that an intervention to improve trust that resulted in an increase of 5-points (moving from agree to strongly agree on all questions) would result in an improvement of 0.1–0.3 in our outcome scores. This improvement is equivalent to the difference in self care scores seen between a 60 year old patient and a 30 year old patient. Additionally, trust was significantly associated with all self care measures while insulin use was not.

Few studies have examined the association of completion of self-care tasks with patient trust. Safran et al surveyed adults employed by the state of Massachusetts using the Primary Care Assessment Survey which includes a section on patient trust [31]. They found that patient trust was significantly associated with patient satisfaction and self-reported adherence to lifestyle changes. Thom et al conducted a longitudinal survey of patients cared for in primary care clinics and found that higher levels of patient trust were associated with greater self-reported adherence to prescribed medications [42]. Our study differs from these by its focus on low-income patients and on measurements of difficulty of self-care in a common, chronic disease.

These results add to growing evidence that patient trust is important to patient outcomes, but are limited by the cross-sectional design of the study. Although the results support the contention that higher levels of trust result in decreased hassles and difficulty in completing self-management task, it is possible that the reverse is true. Longitudinal studies of individuals with newly diagnosed chronic diseases such as diabetes are needed to define the temporal nature of this association. Additionally, our outcome measures did not assess whether patients were actually performing the activities, rather, the instruments were designed to determine the level of difficulty and hassles. It is possible that individuals viewed particular activities as very difficult and having a high degree of hassle and still completed the task. Future studies with measurements of both patient perception and actual completion rates are needed. The shortened trust instrument we used lacked the important dimension of patient-centered care. Patients who feel their concerns are listened to and work in partnership with their health care provider may be more likely to attempt new self care activities. It is likely that the inclusion of this dimension through questions such as the providers' ability to listen to or advocate for the patient would strengthen the association of trust with reduced difficulty in completing self care tasks. We also did not collect information on several important mediators of patient trust and self-care such as the empathy level of the health care provider, educational level of the patient, and patients' overall perceived self-efficacy. These additional factors may provide further elucidation into the relationship of patient trust and ability to complete self care task. Despite these limitations, this study adds to our understanding of the predictors of patient self-management of disease. Further studies comparing strategies for measuring trust, measurement of trust at multiple points in time and the linkage of patient trust with clinical outcomes are needed.

## Conclusions

Trust is an integral component of the doctor-patient relationship. Increasingly, external forces such as changes in reimbursement for medical services and increases in the cost of malpractice insurance place pressure on this relationship [43]. These pressures may erode trust [43]. If trust is associated with self-care tasks such as adherence to medication or lifestyle changes, the turbulent changes in the health care industry may result in worse health outcomes. Efforts to define the impact of trust on patient ability and comfort in performing self-care activities and to identify the modifiable determinants of trust may translate into improved health outcomes, especially for vulnerable populations.

## Competing interests

The author(s) declare that they have no competing interests.

## Authors' contributions

DEB wrote the text and conducted with the statistical analysis.

FB assisted with the statistical analysis and the writing of the paper.

RAB was responsible for selection of the quality of care markers, oversaw data abstraction and assisted with writing the article.

VTD was responsible for data collection and clinic interventions and assisted with editing the article.

RTA was responsible for selection of the quality of life markers and assisted with editing the article.

DCG was responsible for the scientific direction of the study, design, and assisted with editing the article

All authors read and approved the final manuscript

## Pre-publication history

The pre-publication history for this paper can be accessed here:


